# Back and front peripersonal space: behavioural and EMG evidence of top–down and bottom–up mechanisms

**DOI:** 10.1007/s00221-023-06740-4

**Published:** 2023-11-25

**Authors:** Gianna Cocchini, Daniel Müllensiefen, Ruggero Platania, Chiara Niglio, Enrica Tricomi, Laura Veronelli, Elda Judica

**Affiliations:** 1 Psychology Department, Goldsmiths University of London, London, UK; 2 Medizintechnik Group, Institut Für Technische Informatik (ZITI), Heidelberg University, Heidelberg, Germany; 3 Department of Neurorehabilitation Sciences, Casa Di Cura IGEA SpA, Milan, Italy; 4 Department of Psychology, University of Milan-Bicocca, Milan, Italy

**Keywords:** Back space, Rear space, EMG, Blink reflex, Peripersonal space

## Abstract

Previous studies have identified a ‘defensive graded field’ in the peripersonal front space where potential threatening stimuli induce stronger blink responses, mainly modulated by top–down mechanisms, which include various factors, such as proximity to the body, stimulus valence, and social cues. However, very little is known about the mechanisms responsible for representation of the back space and the possible role of bottom–up information. By means of acoustic stimuli, we evaluated individuals’ representation for front and back space in an ambiguous environment that offered some degree of uncertainty in terms of both distance (close vs. far) and front–back egocentric location of sound sources. We aimed to consider verbal responses about localization of sound sources and EMG data on blink reflex. Results suggested that stimulus distance evaluations were better explained by subjective front–back discrimination, rather than real position. Moreover, blink response data were also better explained by subjective front–back discrimination. Taken together, these findings suggest that the mechanisms that dictate blink response magnitude might also affect sound localization (possible bottom–up mechanism), probably interacting with top–down mechanisms that modulate stimuli location and distance. These findings are interpreted within the defensive peripersonal framework, suggesting a close relationship between bottom–up and top–down mechanisms on spatial representation.

## Introduction

Internal representations of space are used to process stimuli located around an individual’s body (Aggius-Vella et al. 2020a; see Serino 2019 for a review). Peripersonal space, referring to the space in front of us, is defined as the portion of space surrounding our body where objects can be grasped and we can interact with the environment (e.g., Rizzolatti et al. 1997). Not surprisingly, the extent of the individual peripersonal space is debatable and can be modulated by different context and interactions with the environment (Sambo et al. 2012a; Serino 2019).

Some authors suggested that a crucial aspect of PPS is related to arm length (e.g., Longo and Lourenco 2007) and different findings indicate that the transition between peripersonal and extrapersonal space is not clearly defined (de Vignemont and Iannetti 2015; Bufacchi and Iannetti 2018) depending on tool use and type of stimuli (Hunley and Lourenco 2018; Longo and Lourenco 2006; Longo et al. 2015; Maravita and Iriki 2004; Canzoneri et al. 2012; Ferri et al. 2015). However, stimuli presented within the peripersonal space can also trigger different responses depending on their perceived nature and whether they are positioned in close proximity to the body (e.g., Spaccasassi et al. 2019), an area known as the ‘Defensive Peri-Personal Space’ *(*DPPS). This is an area within the peripersonal space that represents a ‘safety margin’ surrounding the body (e.g., Graziano and Cooke 2006 p. 845; Sambo and Iannetti 2013; de Vignemont and Iannetti 2015). A fascinating aspect about DPPS is the flexibility of its boundaries and how the magnitude of our response can be modulated by the perceived proximity of stimuli entering this area (e.g., Sambo et al. 2012b).

A series of elegant studies refined the DPPS looking at the magnitude of hand-blink reflex (see Bufacchi and Iannetti 2018 for a review), which is a subcortical reflex elicited by the electrical stimulation of the median nerve on the internal part of the wrist. The electrical stimulation tends to induce a rapid blink response, which is maximised when the hand enters the DPPS and is close to the face (far-near effect; Bufacchi et al. 2015). The authors delimited a bubble-shaped area around the face representing the DPPS where the magnitude of the reflexive response was more intense (see also Versace et al. 2020, 2021). This finding suggests that knowing the location of the hand via proprioceptive information has an impact on a brainstem reflex response. This top–down process is even more evident in a series of studies by Sambo et al. (2012a; see also Bufacchi and Iannetti 2018) where the blink response was modulated by adding or removing a ‘protective’ screen between the participant’s hand and face. These studies also pointed out how the boundaries of this defensive area are malleable, without clear in-or-out zone, and can be affected by a variety of factors (e.g., valence of stimuli) in addition to proximity to the body (Bufacchi and Iannetti 2018).

Surprisingly, very little is known about the peripersonal space behind us (back space) and a few studies have attempted to investigate this portion of the space (e.g., Graziano et al. 1999; Kóbor et al. 2006; Cocchini et al. 2007; Zampini et al. 2007; Occelli et al. 2011; Noel et al. 2015; Aggius-Vella et al. 2018; Aggius-Vella et al. 2019; Aggius-Vella et al. 2020a,b; Aggius-Vella et al. 2022; see also Vallar et al. 1995; Farnè & Làdavas 2002; Viaud-Delmon et al. 2007; Kerkhoff et al. 2006 for studies with clinical population). By means of the hand-blink-reflex paradigm, Bufacchi et al. (2015) did not observe a significantly stronger blink response once the hand was positioned on the back of the head compared to front positions. However, unlike the front space, the rear space is not seen and acoustic stimuli may play a crucial role in the spatial representation of the rear space (Graziano et al. 1999). In fact, adopting an audio-tactile stimulation, Noel et al. (2015) observed that subjective perception of the back space could be modulated during a full-body illusion paradigm. Aggius-Vella et al. (2018) adopted an auditory bisection paradigm to compare participants’ performance on front and back space. The authors concluded that the lack of vision and limited movement in the back space would be responsible for a poorer representation of the back space compared to the front space.

Taken together, these findings suggest that the (defensive) peripersonal space is malleable with no discrete boundaries and it should not be considered a single space but rather as various peripersonal fields modulated by a multitude of factors (Bufacchi and Iannetti 2018). However, although extensive studies have been devoted to exploring the front space, the possible interaction of top–down and bottom–up mechanisms is still unclear, especially for the rear space.

Blinking is a broad response that can be elicited by a multitude of stimuli, including sounds (Esteban 1999; Grosse and Brown 2003; Carlsen et al. 2011; Brown et al. 1991). Since the source of sounds, unlike somatosensory stimuli, is not constrained by the extension of the arm, acoustic stimulation can offer suitable means to explore spatial representation of the back space. Therefore, the scope of this study was to evaluate individuals’ spatial representation for back space compared to front space in an ambiguous environment that offered some degree of uncertainty in terms of both distance and front–back egocentric location of sound sources. In particular, we aimed to consider back space representation by looking at verbal responses about localization of sound sources and blink reflex comparing stimuli in the front–back egocentric space.

## Methods and procedure

### Participants

A priori sample size calculation for behavioural phase (see later) of the study was conducted with G*Power for a 2 × 2 repeated-measures ANOVA (effect size of 0.3; power of 0.8; α of 0.05), which suggested a minimum sample size of 26. A group of 30 healthy volunteers (19 women) with no neurological or psychiatric history entered the study. Their average age was 24.8 (SD = 3.0; range 20–30) with, on average, 18.0 years of formal education (SD = 1.86; range 12–23). According to the Oldfield Questionnaire (Oldfield 1971) score, 3 participants were classified as left-handed and 27 were right-handed. All participants reported normal hearing and right-ear dominance.

Because of the experimental setting described below, an important inclusion criterion was no evidence of claustrophobia 1. The study was approved by Goldsmiths Ethics Committee and participants gave written informed consent before taking part in the study.

### Sound localization task

Each participant sat blindfolded at the centre of an anechoic and soundproof booth of 150 × 160 cm. Two speakers were placed at fixed distances, one directly in front and one behind the participant. Acoustic stimuli were sent from a PC placed outside the soundproof booth and connected via cable with the speakers. Examiners controlled the entire experiment from outside the soundproof room. Acoustic stimuli were developed with Goldwave (digital audio editing software) and consisted of 20 white noise bursts. To render the sound localisation more challenging, the sound pressure was constantly held at 75 dB at ear level and the sound duration was 200 ms. These parameters were selected following a pilot study showing that performance was not at ceiling. The inter-stimulus interval was randomly selected amongst three possible intervals of 5, 10, or 15 s.

**Fig. 1:**
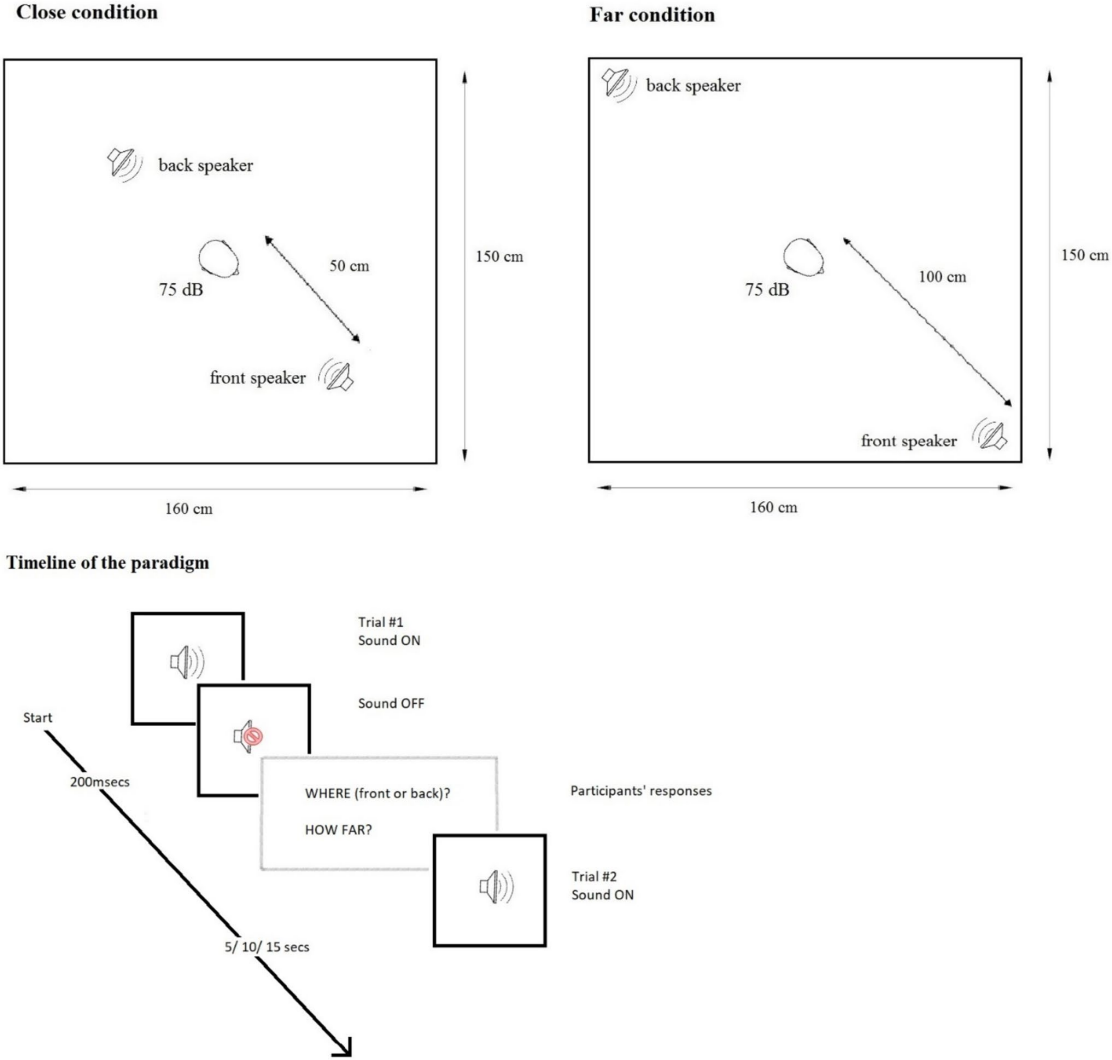
Schematic representation of the setting for each condition and timeline of the paradigm. Paradigm outline not to scale. The time allowed for responses ranged from 5 to 15 s, randomly

The experiment was divided in two blocks of 10 sounds, 5 sounds coming from the front speaker (Front condition) and 5 coming from the back speaker (Back condition). Both speakers were located at 90 cm from the floor (approximately at the height of the participants’ head). Back and Front conditions were randomly presented within each block. In one block, the speakers were located 50 cm from the participant (within reaching space; namely Close condition in our study). In the other block, the speakers were located 1 m from the participant (outside reaching space; namely Far condition in our study; see Fig. 1). The order of the two blocks was counterbalanced across participants. After each block, there was an interval of approximately 5 min during which the participants, with their eyes closed, were carefully guided outside the soundproof room to allow repositioning of the speakers for the next block. Participants were not informed about the change of location of the speakers nor about the number of possible locations. The ambiguity of the sound localization permitted to opt for a block design to allow direct comparisons of back and front sound sources at the same distance; it also minimises sound reflections by minimising the number of objects with hard surfaces (i.e., speakers) in the room.

Behavioural data: Participants were instructed to sit as still as possible and pay attention to brief sounds that were played at different intervals from different positions. Soon after each sound, they provided verbal responses about the location of the sound source (saying “back” or “front”; *perceived location*) and then its distance (*perceived distance*) expressed in their preferred metric (these responses were then all transformed in cm for the analyses). Their verbal responses were audio recorded for later analysis. Importantly, since participants were instructed to keep their eyes closed before entering the testing room and during the entire duration of the experiment, they had no information about the real size of the soundproof room. The sound localisation task lasted about 9 min (approx. 2 min per each block, plus 5 min to reposition the speakers for block 2).

Considering the behavioural data, we aim to explain a) the subjectively perceived location and distance of the sound source and b) the accuracy of participants’ perceived location and distance judgments. As independent variables, we use the actual location (back vs. front) and actual distance (near vs. far). In addition, we test whether perceived, instead of actual, location is a better predictor for perceived distance. This could provide insight into how subjective location and distance judgements are linked.

Electromyographical data: For 26 participants, we also recorded the electromyographic (EMG) activity of the orbicularis oculi muscle bilaterally to measure the blink reflexes in response to the acoustic stimuli. EMG was recorded using two pairs of surface electrodes with the active electrode over the mid lower eyelid and the reference electrode placed laterally to the outer canthus (interelectrode distance 1 cm). The ground electrode was placed on the mastoid. Before positioning the electrodes, skin was accurately scrubbed to reduce skin impedance (Blumenthal et al. 2005). Signals were recorded through a custom-made surface EMG acquisition system performing amplification and digitization at a sampling frequency of 1200 Hz with 24-bit resolution, and saved for offline analysis. A program created with C +  + was designed to synchronise stimuli delivery and EMG data collection. The design of the amplifier removes the DC component with a time constant of 0.1 s. The ADC dynamic was 50 mV due to front-end gain, ADC reference voltage was actually 2.5 V (headroom was ± 2.5 V). Electrodes were positioned before the start of the first block and remained in place for the entire duration of the experiment.

EMG raw signals were bandpass filtered (200–400 Hz, fourth-order Butterworth filter) and cleaned from power line interference (50 Hz notch filter, fourth-order Butterworth). Signal quality was assessed through visual analysis in the time-domain of single blink responses and bad trials (i.e., high levels of noise artifacts or failure of detecting spontaneous blinks) were rejected. For each subject and trial, EMG responses were averaged bilaterally given the symmetrical nature of elicited blinking (Esteban 1999; Blumenthal et al. 1996).

For each trial, we identified the onset and the peak of a blink reflex. The former was identified after signal rectification as the first point exceeding 2 standard deviations of the EMG baseline mean (identified as data in 200 ms before the stimulus delivery; Hodges and Bang 1996); the latter was identified as the peak value in a time window between the delivered stimulus and 200 ms after it (Blumenthal et al. 2005).

EMG responses were then quantified in terms of (i) Onset Latency (latency between the start of each stimulus and blinking onset; (ii) Peak Latency (latency between the start of the stimulus and the peak of blinking; (iii) Peak amplitude. The aforementioned metrics are indeed retained the most representative in quantifying the response to a blinking stimulus from electromyographic signals (Blumenthal et al. 2005; Blumenthal 1996; Berg and Balaban 1999; van Boxtel 2010).

## Results

### Behavioural data

#### Data pre-processing and analysis strategy

All 30 participants completed the Sound localization task for a total of 600 trials across all four conditions. Participants’ responses about distance, originally expressed in their preferred metric, were all converted in the same metric (cm). Individuals’ overall mean estimation of distances across conditions was calculated and two participants (n. 16 and 30) were excluded as their responses were more than 2 SDs^2^ (i.e., 2.72 and 3.34, respectively) from the group mean. A third participant (n. 7) was excluded as she did not provide any response in the Far condition. Therefore, analyses were conducted on the remaining 27 participants for a total of 540 trials across all conditions. Of these, 66 trials (12% across all four conditions with a number of excluded trials ranging from 11 to 22 out of 135 per condition; the median of valid trials per participant was 95% ranging from 55 to 100%) could not be considered for further analysis as they were associated with invalid responses for localization or estimated distance (e.g., “Left”, “Above”, “I don’t know” or there was no response). Therefore, final analyses were conducted on 474 trials across both distance conditions (i.e., 236 for Close and 238 for Far) and across both spatial locations (234 for Front and 240 for Back). Values of d’ were computed at the aggregate level for Close and Far conditions. To by-pass the problem of ceiling or floor effects for some cells, we followed the replacement of data method adopted as in the previous studies (e.g., Baddeley et al. 1999; Brazzelli et al. 1994; Guilford 1954). The average d’ for the Close condition was 0.75 (SD = 1.29), while it was 0.65 (SD = 1.49) for the Far condition; a t test did not show a significant difference between conditions (*p* = 0.423). Overall, 60.55% of the perceived location responses were correct; a one-sample t test analysis indicated that this performance was significantly above chance level (*t* = 26.943; *p* < 0.001). Perceived locations for all conditions are reported in Table 1.

**Table 1:** Perceived location for actual locations and distances

Actual location	Actual distance	Perceived location	*N*	%
**Back**	**50**	**Back**	**78**	**67.2**
Back	50	Front	38	32.8
**Back**	**100**	**Back**	**67**	**54**
Back	100	Front	57	46
*Total back*	*240*			
Front	50	Back	50	41.7
**Front**	**50**	**Front**	**70**	**58**
Front	100	Back	42	36.8
**Front**	**100**	**Front**	**72**	**63.2**
*Total front*			*234*	
*Total (back & front)*			*474*	

Correct responses are highlighted in bold

The distribution of distance responses was heavily skewed (skewness = 2.78, see figure in the supplementary material), and therefore, distance responses were log-transformed.

Location accuracy was scored as a binary variable (0 = incorrect, 1 = correct) and distance accuracy was computed as *|r – a|/a*, where *r* is the distance response in cm and *a* represents the actual distance (50 cm or 100 cm).

All analyses were implemented using mixed effect models using the lmer() function (using a Gaussian error distribution) and the glmer() function (using a binomial error distribution) from the R package lme4. For each analysis, we computed four models that had identical fixed effects (i.e., location and distance and independent variables) but varied in their random effects structure from simple random intercepts for participants to the maximal random effect structure also containing random slopes for both experimental factors (Barr et al. 2013). In particular, we tested models with only random intercepts across participants, models with uncorrelated and correlated random effects for intercepts and slopes for actual location and models with random participant intercepts, and random slopes for actual location and actual distance. These four models differing in their random effect structure were compared on the Bayesian Information Criterion (BIC) and using a likelihood ratio Chi-square test. Once the random effect structure with the best fit to the data was chosen, the fixed effects of this model were then analysed with a type III sum-of-squares (ANOVA) Wald test. In addition, partial *R*^2^ effect sizes are calculated for each fixed effect predictor by refitting the final model using penalised maximum likelihood (via the glmmPQL() function from the R package MASS) and subsequently applying the r2beta() function from the r2glmm package. Further information is provided in the supplementary material file.

**Fig. 2:**
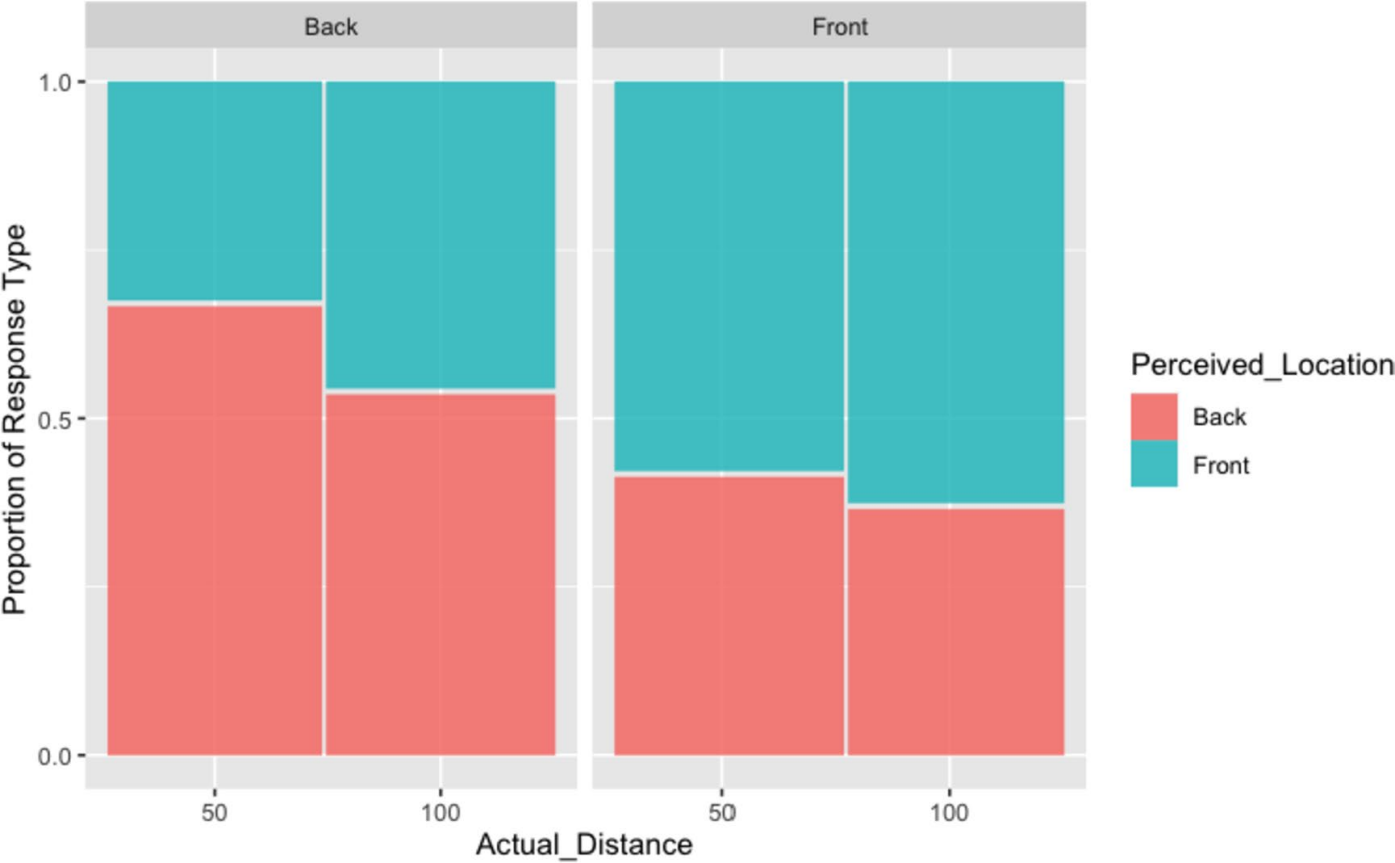
Perceived location for back and front stimuli by experimental conditions (actual location and actual distance)
  In contrast, the model using perceived distance as a dependent variable shows that neither actual location, actual distance nor their interaction influences participant responses significantly. That being said, the effect of actual stimulus direction is approaching the usual significance level and stimuli sounding in the back are perceived as more distant (but *p* = 0.071; see Table 3). This trend is also visible in Fig. 3, as well as the wide confidence intervals around the point estimates.

(i) Modelling perceived location and perceived distance responses
  For modelling perceived location and perceived distance, the models with random participant intercepts and correlated random slopes for actual locations directions each had the best fit (see supplementary material to see model formulae and model comparison results). For the model using perceived location as a dependent variable, Table 2 and the model summary in Fig. 2 indicate that the participant response "front" is significantly associated with front being the actual location but also with the sound coming from the far distance (100 cm).

**Table 2:** Analysis of deviance table (Type III Wald Chi-squared tests) for perceived location

		χ^2^	df	*p*
	*Response: perceived location*			
	(Intercept)	0.0088	1	0.92530
	Actual distance _categorical	13.1265	1	**0.00029**
	Actual location	6.2126	1	**0.01268**
	Actual distance _categorical:Actual Location	1.9981	1	0.15749

		R^2^	95% Conf. interval	
			Lower	Upper
	*Effect*			
1	Model	0.069	0.029	0.139
2	Actual location1	0.039	0.007	0.092
3	Actual distance_catigorical1	0.030	0.004	0.079
4	Actual distance_categorical1: Actual Location1	0.004	0.000	0.032

Significant effects are highlighted in bold

**Table 3:** Analysis of deviance table (Type III Wald Chi-squared tests) for perceived distance

		χ^2^	df	*p*
	*Response: Log_perceived_distance*			
	(Intercept)	411.6023	1	**0.00001**
	Actual distance_catergorical	0.5960	1	0.44011
	Actual location	3.2647	1	0.07079
	Actual distance_categorical:Actual location	0.0223	1	0.88140

		*R*^2^	95% Conf. Interval	
			Lower	Upper
	*Effect*			
1	Model	0.029	0.007	0.085
3	Actual location1	0.027	0.003	0.074
2	Actual distance_categorical 1	0.002	0.000	0.024
4	Actual location_categorical: Actual location1	0.000	0.000	0.017

Significant results are highlighted in bold

**Fig. 3:**
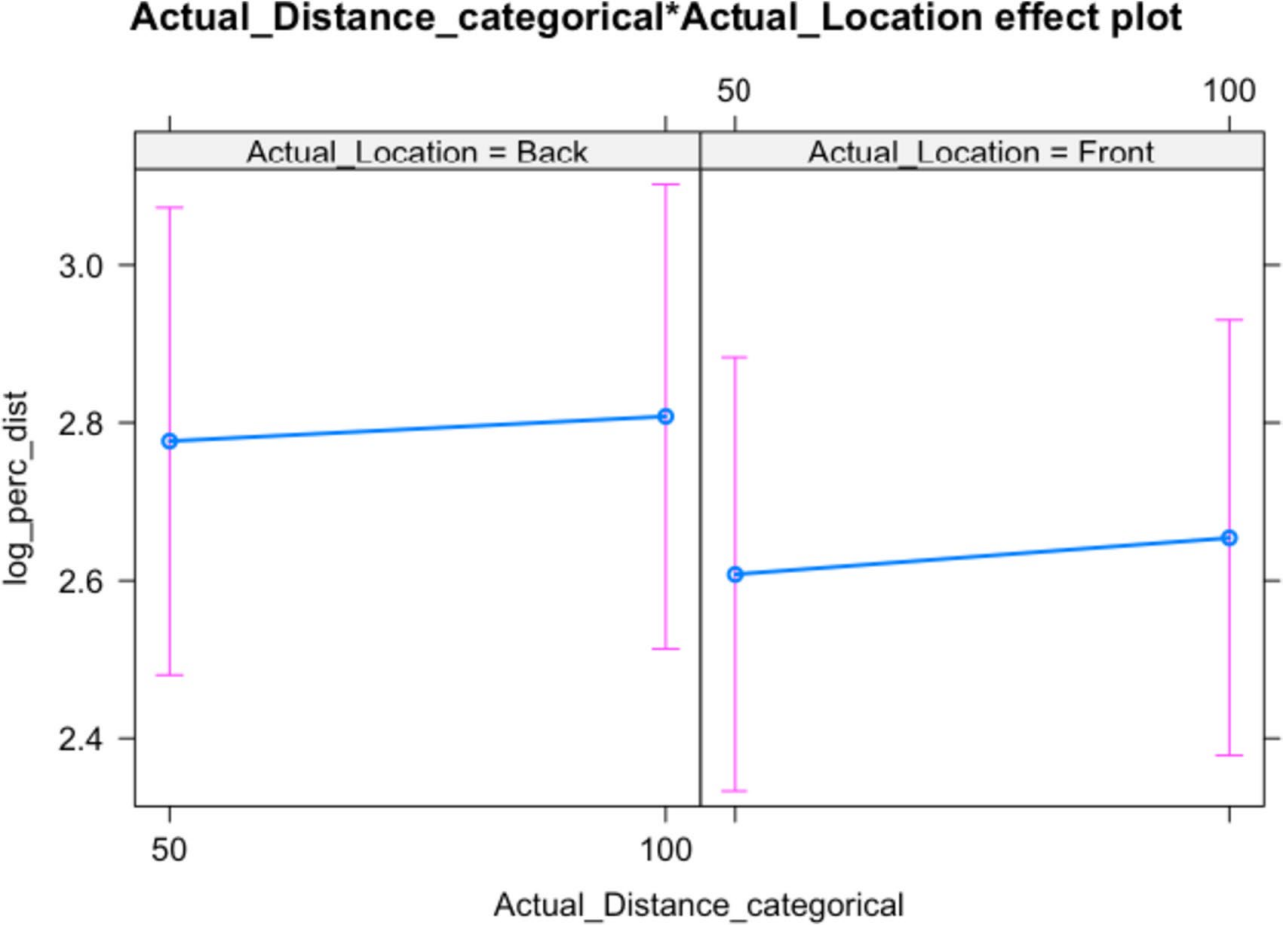
Distance evaluation for back and front stimuli
  Replacing actual stimulus location with perceived stimulus location as a predictor does not improve the modelling of perceived distance judgements. The model with perceived stimulus location as predictor has a BIC = 939, while the model with actual stimulus location has a better BIC = 928. Thus, the accuracy of (log) distance judgements is best modelled with actual location.
(ii) Modelling the accuracy of location and distance responses
  Across all participants, the percentage of accurate responses for perceived location (as ‘front’ or ‘back’) of the sounds was 60.55% (287/474) of which: 60.7% (142/234) for stimuli in the Front, 60.4% (145/240) for stimuli in the Back, 62.7% (148/236) for Close stimuli, and 58.4% (139/238) for Far stimuli. When modelling the accuracy of the perceived location with mixed-effects models (using a binomial error distribution), a model with random intercepts for participants and correlated random slopes for location had the best fit. The summaries of the model tests in Table 4A and B, as well as the corresponding plot (Fig. 4), show that the main effects of actual direction or actual distance do not influence the accuracy of participants’ perceived location, but their interaction does. Stimuli presented at a close distance at the back of the participants are judged most accurately. Stimuli presented from the front and at a far distance are most difficult to judge.

**Table 4:** **A** Analysis of deviance table (Type III Wald Chi-squared tests) for accuracy of perceived location, and **B** model coefficient estimates and significance tests of fixed effects

		χ^2^	df	*p*
(**A**)	*Response: accuracy*			
	(Intercept)	6.2128	1	**0.01268**
	Actual distance_categorical	1.9982	1	0.15749
	Actual location	0.0088	1	0.92529
	Actual distance_categorical: actual location	13.1268	1	**0.00029**

		R^2^	95% Conf. Interval	
			Lower	Upper
	*Effect*			
1	Model	0.034	0.010	0.093
4	Actual distance_categorical: actual location	0.030	0.004	0.079
2	Actual distance_categorical	0.004	0.000	0.032
3	Actual location	0.000	0.000	0.017

		SE	z	*p*
(B)	*Beta*			
(Intercept)	1.4621	0.5866	2.493	**0.01268**
Actual distance_categorical (close)	0.3509	0.2483	1.414	0.15749
Actual location (back)	-0.1396	1.4887	-0.094	0.92529
Actual distance_categorical (close):				
Actual_location (back)	0.8966	0.2475	3.623	**0.00029**

Significant results are highlighted in bold

**Fig. 4:**
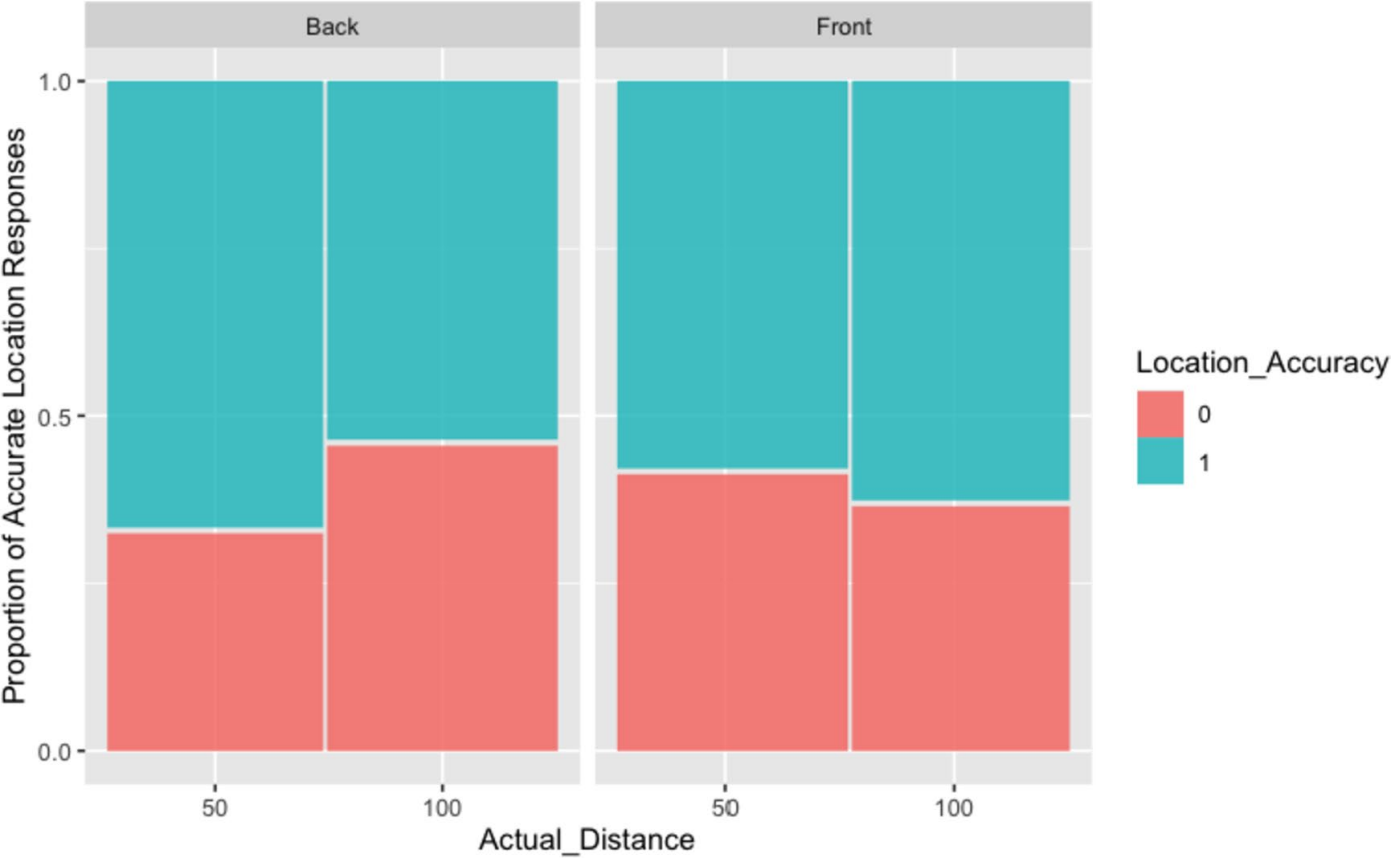
Back–Front location accuracy for Close (50) and Far (100) stimuli
  The means and standard deviations of perceived distance accuracy for each experimental condition are given in Table 5. Because accuracy values are expressed relative to the target distance, this allows for an interpretation in terms of percentage distance from the actual distance of the sound source. Participant perceived distance is much more accurate (about 15%) for near sounds.

**Table 5:** Means and SDs of perceived distance accuracy for each condition

Actual distance	Actual location	Mean	SD
50	Back	0.63	0.27
100	Back	0.77	0.20
50	Front	0.65	0.28
100	Front	0.800	0.14
  When modelling perceived distance, a model with random intercepts for participants and correlated random slopes (across directions and locations) has the best fit. The model-based effects plot in Fig. 5 and the model summary in Table 6 show that only the actual distance—not the direction or their interaction—influence participant responses significantly.

**Fig. 5:**
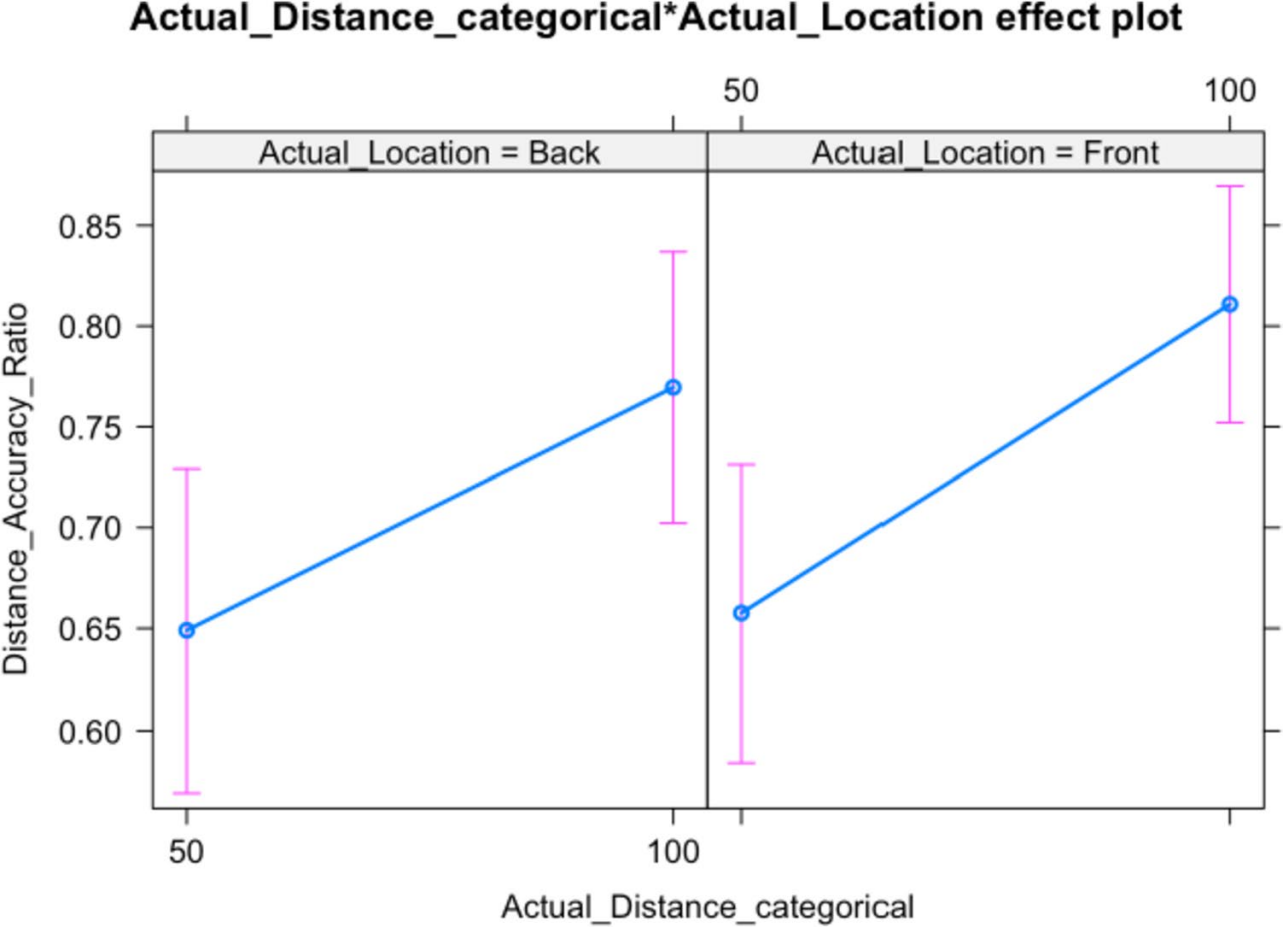
Distance accuracy ratio for Close (50) and Far (100) stimuli

**Table 6:** Analysis of deviance table (Type III Wald Chi-squared tests) for distance accuracy

Response:distance accuracy ratio		χ^2^	df	*p*
	(Intercept)	753.8365	1	**0.0001**
	Actual distance_categorical	23.7161	1	**0.0001**
	Actual location	0.4545	1	0.50020
	Actual distance_categorical: actual location	1.3009	1	0.25410

	Effect	R^2^	95% Conf. interval	
			Lower	Upper
1	Model	0.196	0.128	0.281
4	Actual distance_categorical: actual location	0.19	0.119	0.271
2	Actual distance_categorical	0.008	0.000	0.04
3	Actual location	0.003	0.000	0.029

Significant results are highlighted in bold

#### EMG data

After data pre-processing, as described in the Method section, we quantified/measured the EMG response on 122 trials in terms of (i) Onset Latency, (ii) Peak Latency, and (iii) Peak amplitude. Out of these three variables, only the Peak Amplitude had a distribution that was substantially skewed and required a log-transformation.

- Modelling peak amplitude

**Fig. 6:**
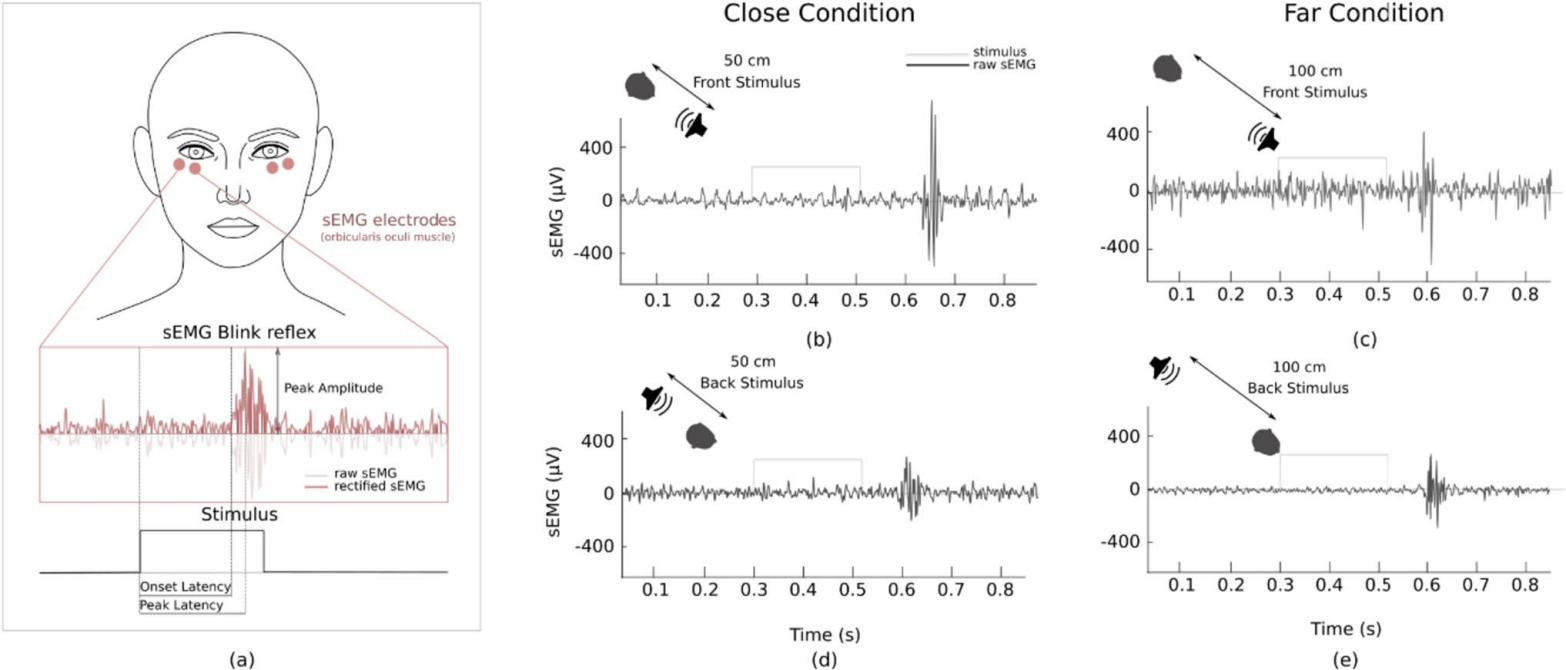
Samples of sEMG blink reflex data (**a**) for Front Close (**b**) and Far (**c**) and for Back Close (**d**) and Far (**e**) conditions

Means and standard deviations for all three dependent variables derived EMG responses are given in Table 7 and illustrated in Fig. 6.

Peak amplitude is best modelled with only random intercepts for participants. However, none of the fixed effect predictors makes a significant contribution towards explaining the EMG peak as Table 8 shows.

**Table 7:** Means and SDs of EMG log-transformation data

EMG measures	Mean	SD
Peak amplitude	5.67	0.74
Peak latency	0.43	0.07
Onset latency	0.34	0.13

**Table 8:** Analysis of deviance table (Type III Wald Chi-squared tests) for peak amplitude

		χ^2^	df	*p*
	*Response: peak amplitude*			
	(Intercept)	855.8569	1	**0.0001**
	Actual distance_categorical	23.9455	1	0.1631
	Actual location	0.243	1	0.2654
	Actual distance_categorical: actual location	1.4708	1	0.2252

		R^2^	95% Conf. Interval	
			Lower	Upper
	*Effec*t			
1	Model	0.053	0.011	0.194
4	Actual Distance_categorical100	0.049	0.001	0.165
2	Actual Distance_categorical100: actual locationF	0.014	0.000	0.098
3	Actual locationF	0.011	0.000	0.092

Significant results are highlighted in bold

Model fit increases when actual stimulus location (BIC = 272) is replaced with perceived stimulus location (BIC = 268) and perceived stimulus location becomes a significant model predictor as Table 9 shows.

**Table 9:** Analysis of deviance table (Type III Wald chi-squared tests) for peak amplitude

		χ^2^	df	*p*
	*Response: peak amplitude*			
	(Intercept)	876.1267	1	**0.0001**
	Actual distance_categorical	1.1404	1	0.2856
	Perceived location	4.3102	1	**0.0379**
	Actual distance_categorical: perceived location	0.318	1	0.5728

		R^2^	95% Conf. Interval	
			Lower	Upper
	*Effect*			
1	Model	0.166	0.067	0.328
4	Perceived LocationF	0.096	0.001	0.231
2	Actual Distance_categorical100	0.031	0.000	0.135
3	Actual Distance_categorical100: Perceived LocationF	0.006	0.000	0.079

Significant results are highlighted in bold

This implies that the EMG peak is better modelled with perceived as opposed to actual location of the sound source.

For modelling the latency between stimulus onset and EMG amplitude peak, a random effect model with random intercepts and random slopes for locations has the best model fit. However, none of the fixed effect predictors reaches the common significance levels in the model. When replacing actual stimulus location with perceived stimulus location, a model fit in terms of the BIC is obtained, but predictors in the resulting model still have non-significant coefficients as Table 10 shows.

**Table 10:** Analysis of deviance table (Type III Wald Chi-squared tests) for peak latency

		χ^2^	df	*p*
	*Response: peak latency*			
	(Intercept)	109.9892	1	**0.0001**
	Actual distance_categorical	2.1490	1	0.1427
	Perceived location	0.0257	1	0.8726
	Actual distance_categorical: perceived location	0.7816	1	0.3767

		R^2^	95% Conf. Interval	
			Lower	Upper
	*Effect*			
1	Model	0.275	0.152	0.435
4	Actual Distance_categorical100	0.230	0.101	0.381
2	Actual Distance_categorical100: Perceived LocationF	0.060	0.002	0.182
3	Perceived LocationF	0.002	0.000	0.062

Significant results are highlighted in bold

A similar picture emerges when the latency between stimulus onset and onset of the EMG amplitude is modelled as a dependent variable. A model with only random intercepts for participants has the closest fit, but neither actual sound location nor actual distance has a significant model coefficient (see Table 11). However, when actual sound location is replaced with perceived sound location, model fit increases and perceived location approaches the common significance level (see Table 11).

**Table 11:** Analysis of deviance table (Type III Wald Chi-squared tests) for onset latency

		χ^2^	df	*p*
	*Response: onset latency*			
	(Intercept)	121.0793	1	**0.0001**
	Actual distance_categorical	1.3645	1	0.2425
	Perceived location	3.5201	1	0.0606
	Actual distance_categorical: perceived location	0.1728	1	0.6777

		R^2^	95% Conf. Interval	
			Lower	Upper
	*Effect*			
1	Model	0.119	0.039	0.277
4	Perceived LocationF	0.044	0.001	0.158
2	Actual Distance_categorical100	0.032	0.000	0.137
3	Actual Distance_categorical100: Perceived LocationF	0.002	0.000	0.062

Significant results are highlighted in bold

Finally, a further analysis considering blink magnitude as a predictive factor did not show a significant contribution (*p* = 0.1991) and a very low effect was associated with it (R2 = 0.003).

## Discussion

Studies evaluating the peripersonal space have indicated that the front space is not uniformly represented and that stimuli presented closer to the body may trigger stronger responses as they fall in a defensive graded field (Bufacchi and Iannetti 2018). However, much less is known about the rear space where accurate location of stimuli, sounds in particular, would be crucial from an evolutionary perspective (Kolarik et al. 2016). By means of a sound localization paradigm, our study aimed to establish whether participants hold a different spatial representation for front and back space and whether acoustic stimuli at different distances from the body could trigger stronger (defensive) blink responses.

A natural methodological limitation to evaluate spatial representation with sounds at various distances is sound reverberation and the difference of the sound level reaching the individual’s ear (Zahorik and Wightman 2001), which is “the most reliable auditory distance cue” available to participants (Aggius-Vella et al. 2022; p. 4). To enhance some degree of uncertainty about localisation, the study was run in a soundproof environment and the sound loudness was maintained constant at the level of the participant’s ear. Clearly, some minor differences amongst the perceived sounds could have guided, at least in part, the participants’ response about location. Indeed, the localisation task proved to be rather challenging, with an overall localisation accuracy above chance of 60%, comparable for both front and back stimuli; an ambiguity that became crucial for further analyses. Importantly, the lack of significant difference between front and back discrimination suggests that our participants did not show an overall response bias for either position.

The first set of analyses with behavioural data was conducted considering the effect of actual location and distance of acoustic stimuli on subjective evaluation of location and perceived distance of the stimuli. Findings indicated that participants’ perceived location (front vs back) of sounds was influenced by the actual location and distance of the stimuli, but we also observed a significant tendency to perceive stimuli originated in more distant locations as coming from the front. It seems, therefore, that participants tended to correctly locate the source (front vs back) of the sounds above chance level, but their more frequent front–back discrimination error was to misallocate distant back sounds to the front space. This type of error trend may have mitigated possible effects during the distance estimation of the stimuli as an incorrect location of a stimulus will inevitably affect all spatial references, making distance judgments meaningless. Thus, it was crucial to take into account the accuracy of perceived location in a second set of analyses.

When the accuracy for the perceived location of stimuli was considered in the mixed effect models, we observed an interaction between actual location and actual distance. The interaction suggested that participants were more accurate in locating back sounds in the close condition and more accurate in locating front stimuli in the far condition. Sounds in the far back condition tended to be misallocated in the front space. It is important to note that, overall, participants’ perceived distance of sounds was about 15% more accurate for close stimuli than far stimuli. This result may be due, at least in part, to a general underestimation of distances in auditory representation for sounds located at the edge or beyond the reaching space (e.g., Zahorik et al. 2005; Kearney et al. 2012), though this aspect is still not well understood (Kolarik et al. 2016).

Taking these findings together, it seems that stimuli perceived as close to the body, regardless of their actual location, were more likely to be allocated in the back space and their distance was generally estimated accurately. On the contrary, stimuli perceived as distant, regardless of their actual location, were more likely to be subjectively allocated in the front and their distance considerably underestimated, as if the front space was compressed. The perceived distance of the sounds is not per se important, but its relationship with the subjective front or back space is interesting.

Based on studies recording blink responses for stimuli located in the front space (e.g., Graziano and Cooke 2006; Bufacchi and Iannetti 2018), we would expect that stimuli perceived as close to the body would induce stronger blink responses than stimuli perceived as distant. However, little is known about the back space and a recent study by Bufacchi et al. (2015) did not observe the expected strong (defensive) blink reflex when the hand was positioned on the back of the head. This may be due to the fact that the back space is rarely explored with the hand and acoustic stimuli seem more informative for it. Considering our EMG data, we did not find a significant impact of actual location. Like our behavioural findings, the blink responses were better explained by perceived location rather than actual location. In other words, the actual location or distance of sounds did not lead to different blink responses. We only observed a modulation of the blink response when the perceived location of stimuli was considered.

At this point, it is important to note that the verbal (location and distance) responses inevitably occurred after the fast process linked to blink reflex; therefore, these findings indicate that the mechanisms also underling the blink reflex may have guided, possibly unconsciously, the participant’s front–back discrimination and verbal response. In particular, we recorded higher peak amplitudes in response to stimuli that were later verbally localised in the front. This implies that the participants tended to locate those stimuli that caused a more intense blink response in the front space. It is unclear what determined a different strength of the blink response in the first place and we do not exclude that more complex mechanisms may play a role. For example, the sound level may have not been perfectly equivalent across conditions as the shape of the pinna or minimal movements of the head may have played a role in this. Other factors may have enhanced reflex responses (Kolarik et al. 2016) as blink responses did not add predictive value to determine sound location per se. It is more likely that common factors underlying reflex responses and spatial location may have interacted and guided the final behaviour. It seems, therefore, that participants’ responses, clearly dominated by auditory sensory information, can be further modulated by an interaction of top–down processes (e.g., Bufacchi and Iannetti 2018; Versace et al. 2020; 2021) and additional bottom–up mechanisms, which dictate blink response magnitude. Within the ‘defensive graded field’ framework (Bufacchi et al. 2015; Bufacchi and Iannetti 2018), we could speculate that a strong blink reflex may be associated with a more threatening situation (Versace et al. 2021), which in turn requires more attention and an individual may ‘prefer’ to represent it in the front space. Perrott et al. (1990) claimed that the primary function of sound localization consists of providing information to allow individuals to move their eyes and “bring the fovea into line with an acoustically active object” (p. 214). It is indeed very common to turn our head (and body) toward a stimulus that may represent a potential threat or that requires more attention. In doing this, the egocentric front–back environment is completely and continuously reshaped and the stimulus is ‘moved’ in the front representational space supported by additional modalities (in particular vision; Aggius-Vella et al. 2022). Even if our participants were blindfolded, the alignment of auditory and visual spatial representations is constantly updated (Lewald 2013; Kolarik et al. 2016). In line with the hypothesised ‘supremacy’ of front space also for auditory representation, Aggius-Vella et al. (2022) found that sighted people were more accurate than blind people in localising sounds in the front space, suggesting that visual experience plays a crucial role in accurately representing auditory spatial representation for the front space. In this case, we may expect that stimuli requiring increasing attentional resources, such as those signalling a potential ongoing threat, may be represented in the front space where spatial organization is also refined by vision and it overlaps with the hand-action area.

### Limitations and alternative explanations

A very different outcome may be expected for stimuli well outside the peripersonal space where any potential threats are much less salient. Our ‘relatively’ far condition was probably just outside the reaching space (Kolarik et al. 2016) and we could not investigate the far extrapersonal condition due to the size constraint of the soundproof booth. Similarly, our ‘relatively’ close condition was just within the reaching areas and stimuli even closer to the face, and well inside the ‘defensive graded field’, could trigger more intense responses. Future studies may attempt to by-pass this limitation using advanced acoustic methods to mimic different distances on headphones. Moreover, the block design did not lead to a ceiling performance and both location and distance evaluations of the sound source proved to be challenging. However, it would be interesting to run a similar study with multiple sources in larger soundproof rooms to better refine the representation of egocentric space. Finally, future studies may aim to systematically manipulate, within the same experiment, top–down and bottom–up processes by providing information about the actual front–back position of the stimuli in advance (top–down) and manipulating the sound intensity at the same distance (bottom–up). To this aim, it would be interesting to mimic the hand-blink-reflex paradigms where proprioceptive information provides the actual location of their stimulated body part. With acoustic stimuli, top–down information could be manipulated by providing congruent and incongruent information about the egocentric location (front or back) of the acoustic source. Manipulation of these factors may provide further interesting information on sound localization and, more crucial to our study, a more detailed representation of egocentric space.

## Conclusion

In line with the current literature, our findings underlined the crucial role of top–down processes that lead individuals to locate and respond to stimuli in different locations around the body, including the back space. We also suggest that bottom–up mechanisms, common to the blink reflex and additional to those directly linked to acoustic sensory information, can also play a crucial role on later and higher cognitive decisions, offering a more complex scenario where higher cognitive processes and physiological responses concur to create the final subjective representation of the peripersonal space.

## Data Availability

Data accessibility: Supplementary material, anonymized data and scripts for the analyses are publicly available at https://osf.io/8vmq6/.

## Footnotes

1 To not draw attention to the question about claustrophobia, which may have provided clues on the possible stimuli distances, this information was requested as part of a list of standard questions about general health and possible allergies to material used to record the EMG signal.

2 An a-priori exclusion of outliers was decided to rather narrow the generalisability of findings than increasing the risk of false-negative results. On a single t test analysis (Crawford and Garthwaite 2007), both outliers’ distance estimations significantly (*p* < .05) differed from the group mean.
